# Increased Acetylcholine Levels and Other Brain Effects in 5XFAD Mice after Treatment with 8,14-Dihydroxy Metabolite of Efavirenz

**DOI:** 10.3390/ijms23147669

**Published:** 2022-07-11

**Authors:** Natalia Mast, Yong Li, Irina A. Pikuleva

**Affiliations:** Department of Ophthalmology and Visual Sciences, Case Western Reserve University, Cleveland, OH 44106, USA; nvm2@case.edu (N.M.); yxl665@case.edu (Y.L.)

**Keywords:** efavirenz, CYP46A1, 8,14-dihydroxyefavirenz, Alzheimer’s disease, cholesterol metabolism, acetylcholine, acetyl-CoA

## Abstract

Efavirenz (EFV), an FDA-approved anti-HIV drug, has off-target binding to CYP46A1, the CNS enzyme which converts cholesterol to 24-hydroxycholesterol. At small doses, EFV allosterically activates CYP46A1 in mice and humans and mitigates some of the Alzheimer’s disease manifestations in 5XFAD mice, an animal model. Notably, in vitro, all phase 1 EFV hydroxymetabolites activate CYP46A1 as well and bind either to the allosteric site for EFV, neurotransmitters or both. Herein, we treated 5XFAD mice with 8,14-dihydroxyEFV, the binder to the neurotransmitter allosteric site, which elicits the highest CYP46A1 activation in vitro. We found that treated animals of both sexes had activation of CYP46A1 and cholesterol turnover in the brain, decreased content of the amyloid beta 42 peptide, increased levels of acetyl-CoA and acetylcholine, and altered expression of the brain marker proteins. In addition, male mice had improved performance in the Barnes Maze test and increased expression of the acetylcholine-related genes. This work expands our knowledge of the beneficial CYP46A1 activation effects and demonstrates that 8,14-dihydroxyEFV crosses the blood–brain barrier and has therapeutic potential as a CYP46A1 activator.

## 1. Introduction

CYP46A1 (cytochrome P450 46A1 or the CNS-specific cholesterol 24-hydroxylase) controls cholesterol elimination and turnover in the brain and, thereby, a variety of brain processes [1,2]. CYP46A1 plays a unique role in the brain and is emerging as a therapeutic target for treatment of different neurodegenerative (e.g., Alzheimer’s, Huntington’s, prion, Niemann–Pick type C1, and spinocerebellar ataxia) as well as non-degenerative brain diseases (e.g., glioblastoma and depression). The therapeutic potential of CYP46A1 was revealed by studies of various animal models, in which increases in CYP46A1 activity were achieved either by gene therapy or pharmacologic activation [3,4,5,6,7,8,9,10,11,12,13]. The latter is exemplified by small dose EFV (0.1 mg/kg body weight), which was administered to 5XFAD mice, a model of rapid amyloidogenesis in the brain [14]. EFV treatment improved animal performance in behavioral tests and led to treatment paradigm-specific changes in the brain amyloid β (Aβ) load, macrophage and astrocyte activation, and expression of important synaptic proteins [6,7,15]. These effects were linked to CYP46A1 activation and increased brain cholesterol turnover [16].

Originally, EFV was developed as an inhibitor of the reverse transcriptase enzyme, which transcribes the HIV RNA into DNA. Then, we discovered that EFV has an unanticipated interaction with CYP46A1 and activates this enzyme at small doses in mice [17] and humans (ClinicalTrials.gov NCT03706885). Studies with purified CYP46A1 revealed that EFV binds to the allosteric site on the P450 cytosolic surface, which is away from the enzyme active site inside the protein molecule [18]. Moreover, we established that aside from EFV, which is xenobiotic, purified CYP46A1 is allosterically activated in vitro by different endogenous compounds such as L-Glu, acetylcholine (Ach), and other neurotransmitters [19]. The site for the neurotransmitter binding on CYP46A1 was identified and found to be different from that for EFV, although in a close spatial proximity, i.e., in the region separated from the EFV binding site by a surface loop consisting of nine amino acid residues [19]. Remarkably, simultaneous incubations of CYP46A1 in vitro with a xenobiotic (EFV) and endobiotic (L-Glu) activator led to a higher P450 activation than in the incubations with the individual activators, meaning that they were synergistic [19].

In both mice and humans, EFV (has the *(S)* stereocenter) is mostly cleared by the liver, leading to the production of different hydroxymetabolites, which are inactive against HIV. The major metabolite is *(S)*-8-hydroxyEFV, while *(S)*-7-hydroxyEFV, 7,8-dihydroxyEFV, and *(S)*-8,14-dihydroxyEFV represent the minor products of the phase 1 EFV biotransformations [20,21,22,23,24]. EFV activates CYP46A1 at a very small dose (0.1 mg/kg of body weight in mice or 50–200 mg/day in humans). Hence, we investigated whether its hydroxymetabolites could activate CYP46A1 as well. Studies in vitro using purified CYP46A1 showed that indeed, 7-hydroxyEFV, 8-hydroxyEFV, 7,8-dihydroxyEFV, and 8,14-dihydroxyEFV (either as *(S)*-isomer or racemic mixture) activated the P450, and it was the hydroxylation position rather than chirality that determined the CYP46A1 activation pattern (the extent of the P450 activation, the shape of the metabolite concentration dependence curve, and the allosteric site for binding) [25]. The latter was either the site for EFV (7,8-dihydroxyEFV), neurotransmitter (8,14-dihydroxyEFV), or both 7-hydroxyEFV and 8-hydroxyEFV [25].

Herein, we conducted in vivo evaluation of (rac)-8,14-dihydroxyEFV (8,14-dihydroxyEFV) as this EFV metabolite binds to the neurotransmitter allosteric site and elicits the highest CYP46A1 activation in vitro. We found that when used at the same dose and treatment paradigm as EFV, 8,14-dihydroxyEFV activated CYP46A1 in the brain of 5XFAD mice and affected various brain processes. The present study reveals similarities and differences in the in vivo (brain) effects of EFV and 8,14-dihydroxyEFV and justifies similar investigations of other EFV hydroxymetabolites

## 2. Results

### 2.1. Brain Sterols

**Figure 1:**
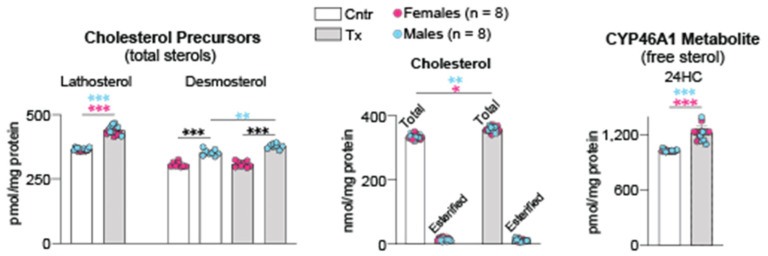
8,14-DihydroxyEFV effect on sterol content in the brain. Data represent the mean ± SD of the measurements in individual 5XFAD mice (8 female and 8 male). * *p* ≤ 0.05; ** *p* ≤ 0.01; *** *p* ≤ 0.001 as assessed by two-way ANOVA with Tukey’s multiple comparison test. The asterisk color indicates significance between female mice (pink), male mice (blue), or male-female animals (black). Cntr, control mice; Tx, treated mice.

Cholesterol does not cross the blood–brain barrier [26]. Therefore, the steady state levels of brain cholesterol represent a balance between in situ biosynthesis and enzymatic elimination via CYP46A1-catalyzed cholesterol 24-hydroxylation [27]. We quantified brain lathosterol and desmosterol (two cholesterol precursors and markers of cholesterol biosynthesis in neurons and astrocytres, respectively [28]), cholesterol, and 24-hydroxycholesterol (24HC, the product of CYP46A1 activity). A 6-month treatment of 5XFAD mice with 8,14-dihydroxyEFV increased their brain levels of lathosterol by 20% (in both female and male mice) and desmosterol by 7% (only in male mice) (Figure 1). The levels of the total brain cholesterol were increased by 7% (in both female and male mice), and the production of 24HC was increased by 18% in both sexes. Thus, similar to EFV [7], 8,14-dihydroxyEFV likely crossed the blood–brain barrier from the systemic circulation and activated CYP46A1 in the brain. This activation led, in turn, to a compensatory increase in the brain cholesterol biosynthesis and, hence, cholesterol turnover.

### 2.2. Brain Aβ Content

**Figure 2:**
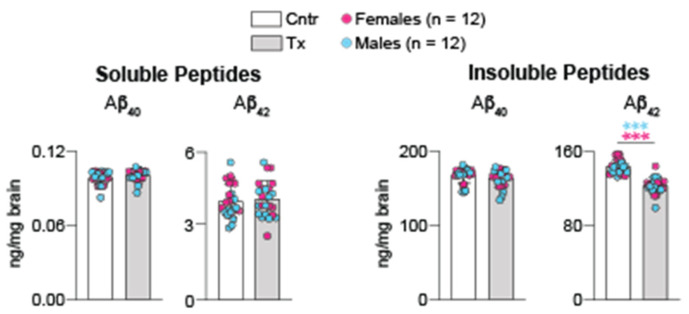
8,14-DihydroxyEFV effect on the brain Aβ peptides. Data represent the mean ± SD of the measurements in individual 5XFAD mice (12 female and 12 male). *** *p* ≤ 0.001 as assessed by two-way ANOVA with Tukey’s multiple comparison test. The asterisk color indicates significance between female mice (pink) and male mice (blue). Cntr, control mice; Tx, treated mice.

Extracellular deposits of Aβ peptides is a hallmark of Alzheimer’s disease (AD) [29]. Brain homogenates were used to measure the overall content of soluble and insoluble Aβ_40_ and Aβ_42_ peptides by ELISA. In 5XFAD mice treated with 8,14-dihydroxyEFV, no changes were documented in the levels of soluble and insoluble Aβ_40_ peptides and soluble Aβ_42_ peptide (Figure 2). Yet, the levels of insoluble Aβ_42_ peptide, the initial amyloid species deposited into Aβ plaques in AD [30], were decreased by 15%, a beneficial effect of treatment. In contrast, when EFV was used in a similar treatment paradigm, the drug did not alter the brain Aβ content (Aβ_40_ and Aβ_42_). Rather, there were local decreases by 17–20% in the number and area of dense core Aβ plaques in mouse cortex and hippocampus as assessed by a histochemistry stain with Thioflavin S [7]. Thus, as compared to EFV, 8,14-dihydroxyEFV seemed to have a more robust effect on the brain Aβ load.

### 2.3. Behavioral Assessments

**Figure 3:**
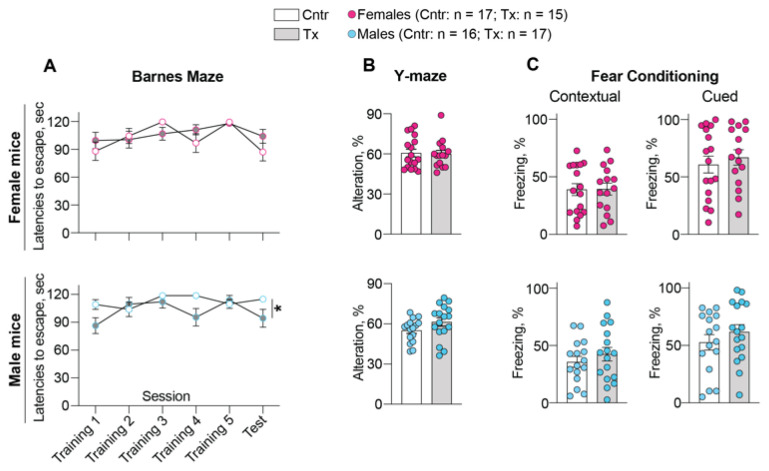
8,14-DihydroxyEFV effect on performance of 5XFAD mice in behavioral tasks: (**A**), Barnes Maze test; (**B**), Y-maze test; and (**C**), fear conditioning tests. Data represent the mean ± SEM of the measurements in individual mice (17 control female mice, 15 treated female mice, 16 control male mice, and 17 treated male mice). Statistical significance was assessed by two-way repeated measures ANOVA with Bonferroni correction (the Barnes Maze test) and a two-tailed unpaired Student’s *t*-test (the Y-maze test and fear conditioning tests). * *p* = 0.03 was only detected between the session number and treatment factor in the Barnes maze test for male mice. Cntr, control mice; Tx, treated mice.

The tests were either similar (the Barnes Maze (BM) instead of Morris Water Maze) or identical (the Y-maze and fear memory tests) to those used in studies of EFV [6,7]. In the BM test, which evaluates the long-term spatial memory, only 8,14-dihydroxyEFV-treated male mice appeared to be affected by showing a trend to learning during some training sessions (sessions 1 and 4, *p* = 0.09 and 0.08, respectively), as indicated by a decrease in the mean latency to escape the plate (Figure 3A). A trend to learning was also observed in the test session (*p* = 0.17), conducted 48 h after the last training, and statistical significance (*p* = 0.03) was detected between the session number and treatment factor. Conversely, no changes in learning, even at the level of a trend, were documented in the treated female mice either during training or the test sessions.

Similarly, sex-based differences were documented in the 8,14-dihydroxyEFV treatment effect in the Y-maze test, which assesses the short-term spatial memory (Figure 3B). Only the treated male mice showed a trend (*p* = 0.12) to improved performance as indicated by an increase in spontaneous alteration rate. Yet, no treatment effect was observed for the treated female mice.

Only in the fear conditioning memory tests, male and female mice responded similarly, i.e., did not show any change in both contextual and cued memory tests as indicated by freezing time (Figure 3C). Thus, as compared to EFV-treated mice, which showed improved learning in the Morris Water Maze test and contextual fear conditioning test with no sex-based differences [7], 8,14-dihydroxyEFV elicited a much weaker effect on mouse behavior and only in male mice.

### 2.4. Brain Acetyl-CoA Levels

**Figure 4:**
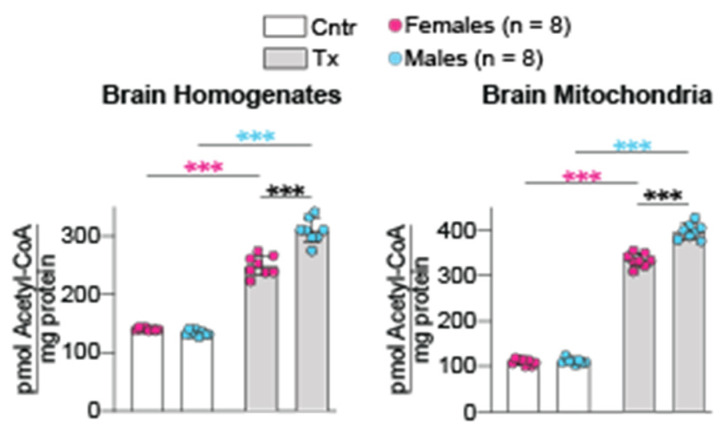
8,14-DihydroxyEFV effect on the brain acetyl-CoA levels. Data represent the mean ± SD of the measurements in individual mice (8 female mice and 8 male mice). *** *p* ≤ 0.001 as assessed by two-way ANOVA with Tukey’s multiple comparison test. The asterisk color indicates significance between female mice (pink), male mice (blue) or male-female animals (black). Cntr, control mice; Tx, treated mice.

Acetyl-CoA is an important endogenous compound, which controls many cellular processes [31]. Previously, we linked the brain acetyl-CoA levels (in both brain homogenates and mitochondria) to CYP46A1 activation by EFV and CYP46A1-mediated sterol flux through the plasma membranes [32]. Herein, we investigated whether CYP46A1 activation by 8,14-dihydroxyEFV had a similar effect on the brain acetyl-CoA levels. Indeed, in both brain homogenates and mitochondria, the acetyl-CoA levels were increased in female and male 5XFAD mice after the 8,14-dihydroxyEFV administration (Figure 4). Importantly, the treated male mice had a higher increase in the homogenate and mitochondrial acetyl-CoA content than the treated female mice (2.3-fold vs. 1.8-fold and 3.6-fold vs. 3.1-fold, respectively), despite control 5XFAD mice having no sex-based differences in the absolute metabolite levels. Thus, like EFV, 8,14-dihydroxyEFV increased the acetyl-CoA content in brain homogenates and mitochondria of 5XFAD mice.

### 2.5. Brain Ach Levels

**Figure 5:**
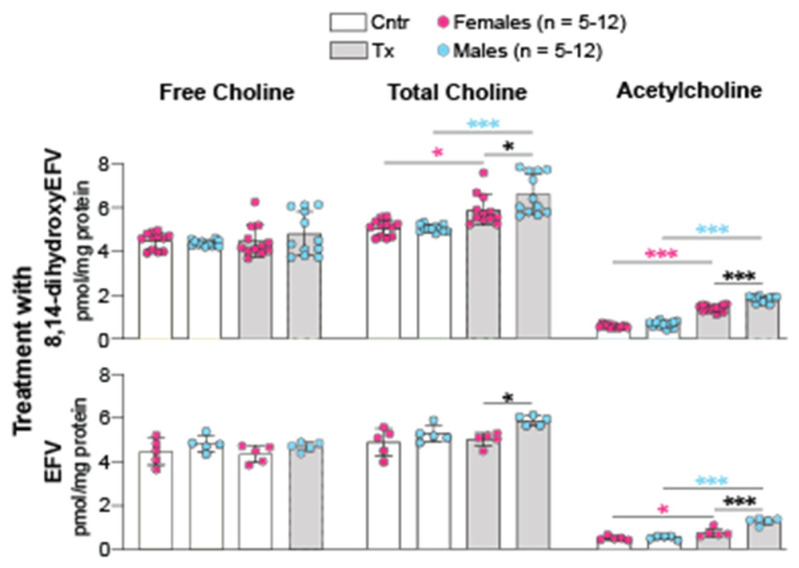
8,14-DihydroxyEFV and EFV effects on the brain Ach levels. Data represent the mean ± SD of the measurements in individual mice (5–12 female mice and 5–12 male mice). *, *p* ≤ 0.05; ***, *p* ≤ 0.001 as assessed by two-way ANOVA with Tukey’s multiple comparison test. The asterisk color indicates significance between female mice (pink), male mice (blue) or male-female animals (black). Cntr, control mice; Tx, treated mice.

Normally, the brain levels of Ach, an excitatory neurotransmitter, depend on the availability of choline and acetyl-CoA, which are used as the substrate and acetylating agent, respectively, in a single step enzymatic reaction yielding Ach [33]. The brain free and total (a sum of free choline and Ach) choline contents were quantified, and then the Ach levels were calculated as a difference between the two measurements. 8,14-DihydroxyEFV did not affect the brain free choline levels, yet increased the total choline levels, thus suggesting that there is an increase in the Ach levels (Figure 5). Like with acetyl-CoA, this increase was higher in the treated male mice than the treated female mice (2.7-fold vs. 2.5-fold).

The acetyl-CoA-Ach connection was not investigated in EFV treatment. Therefore, the brain free and total choline levels were measured in EFV-treated vs. control 5XFAD mice from the same cohort that was used previously for the acetyl-CoA quantifications [32]. The Ach levels were increased after EFV treatment (1.5-fold and 2.5-fold in female and male mice, respectively), although to a lower extent than in 8,14-dihydroxyEFV-treated mice (Figure 5). The absolute neurotransmitter concentrations were also lower after EFV treatment when compared to 8,14-dihydroxyEFV treatment (0.7–1.2 vs. 1.4–1.8 pmol/mg protein, repectively). Nevertheless, in both EFV and 8,14-dihydroxyEFV-treated mice, increased production of acetyl-CoA led to increased production of Ach. This is a new and important insight into the beneficial treatment effect as the brain Ach levels are decreased in AD due to selective degeneration of cholinergic neurons [34]. The latter leads to impaired cholinergic neurotransmission and contributes to cognitive decline in AD [35].

### 2.6. Brain Expresion of Ach-Related Genes

**Figure 6:**
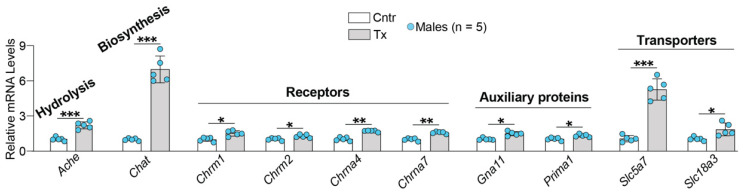
8,14-DihydroxyEFV effect on brain expression of Ach-related genes. Data represent the mean ± SD of the measurements in five individual male mice. * *p* ≤ 0.05; ** *p* ≤ 0.01; *** *p* ≤ 0.001 as assessed by a two-tailed unpaired Student’s *t*-test. Cntr, control mice; Tx, treated mice.

To gain further mechanistic insight into the acetyl-CoA-Ach link, brain expression of the major Ach-related genes was studied after 8,14-dihydroxyEFV administration. Only male mice were used as this sex showed the highest increase in Ach levels (Figure 5). Several groups of genes were examined, and of them, all showed increased expression in 8,14-dihydroxyEFV-treated vs. control 5XFAD mice (Figure 6). The two highest increases were in the expression of *Chat* (7-fold) and *Slc5a7* (5.2-fold), which encode choline O-acetyltransferase and the high affinity choline transporter 1, respectively. The latter imports choline from the extracellular space into cholinergic neurons, and the former uses choline and acetyl-CoA to synthesise Ach. While both acetyl-CoA and choline do not saturate CHAT in vivo [33], choline uptake from the extracellular space is the rate-limiting step in acetylcholine synthesis [36,37]. The third highest increase (2.2-fold) was in the expression of *Ache* encoding acetylcholine esterase, the enzyme that rapidly hydrolyzes Ach at neuromuscular junctions and brain cholinergic synapses and terminates signal transmission [38]. An increase in expression of *Ache* was much lower than that of *Chat* and *Slc5a7*, thus suggesting that increased Ach levels in 8,14-dihydroxyEFV-treated 5XFAD mice could be due to increased production of acetyl-CoA coupled to increased expresion of *Chat* and *Slc5a7*.

Unlike *Chat*, *Slc5a,* and *Ache,* the expression of the genes encoding several abundant receptors (muscarinic *Chrm1* and *Chrm2*) or receptor subunits for Ach (nicotinic *Chrna4* and *Chrna7*) [39] was increased only moderately (from 1.3-fold to 1.7-fold) (Figure 6). Similarly, a moderate increase (from 1.3-fold to 1.8-fold) was observed in the expression of the genes for two auxiliray proteins (*Gna11* and *Prima1*) as well as *Slc18a3* (Figure 6), the Ach transporter from the cytosol into presynaptic vesicles for release into the synaptic space [40]. *Gna11* encodes subunit 11α of the G protein (G_q/11_), which interacts with CHRM1, CHRMR3, and CHRM5 and mediates receptor signaling [41]. *Prima1* encodes the protein which organizes ACHE into tetramers and anchors the enzyme to the membrane of neuronal synapses [42]. Increased expression of proteins involved in the brain cholinergic neurotransmission was consistent with increased expression of CHAT, the most specific indicator for monitoring the functional state of cholinergic neurons in the central and peripheral nervous systems [43]. Thus, treatment with 8,14-dihydroxyEFV potentially had a beneficial effect on the brain cholinergic signaling.

### 2.7. CYP46A1 Activation In Vitro

**Figure 7:**
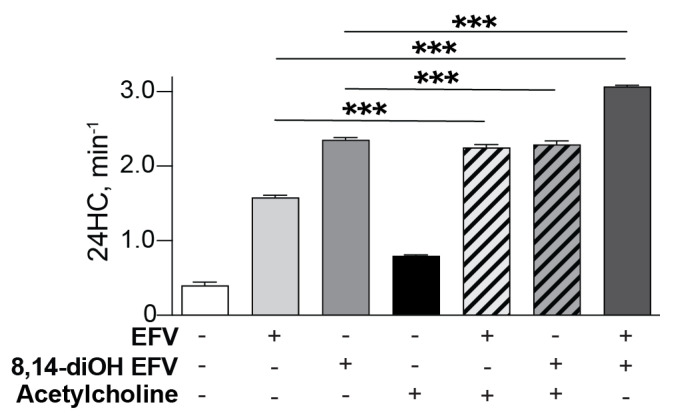
CYP46A1 activation in vitro in the presence of different compounds. CYP46A1 activity is presented as nanomoles of 24-hydoxycholsterol (24HC) formed per nmole of CYP46A1 per min. The results are the mean ± SD of the measurements from the three independent experiments. *** *p* ≤ 0.001 as assessed by a two-tailed unpaired Student’s *t*-test. 8,14-diOH EFV, 8,14-dihydroxyEFV.

Only L-Glu was previously investigated in vitro in co-incubations of EFV or 8,14-dihydroxyEFV with CYP46A1 and found to increase and decrease CYP46A1 activity, respectively, as compared to the individual enzyme incubations with each compound [19,25]. We conducted similar experiments with Ach, EFV and 8,14-dihydroxyEFV using purified CYP46A1 (Figure 7). In co-incubations of EFV and Ach, the CYP46A1 activation was increased as compared to the incubations with the individual activators, i.e., was consistent with the two activators binding to different allosteric sites and eliciting a synergistic effect. Yet, in co-incubations of 8,14-dihydroxyEFV and Ach, the CYP46A1 activity was essentially the same (only slightly lower) as in the incubation with 8,14-dihydroxyEFV. This result was consistent with the two activators competing for the same allosteric site, and the 8,14-dihydroxyEFV binding being preferential. Thus, if L-Glu and Ach activate CYP46A1 in vivo, the P450 activation by 8,14-dihydroxyEFV, but not EFV, could be decreased by L-Glu and unaffected by Ach and become similar to that induced by EFV, in agreement with the data on in vivo CYP46A1 by EFV [7] and 8,14-dihydroxyEFV (Figure 1).

### 2.8. Brain Levels of Various Marker Proteins and CYP46A1

**Figure 8:**
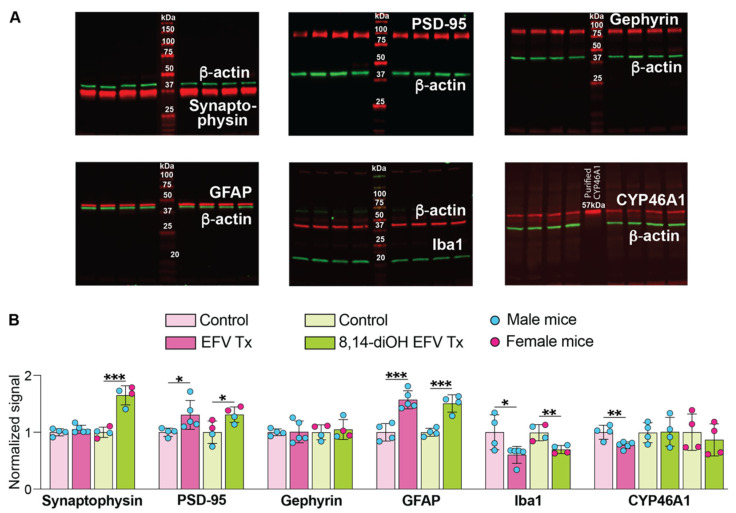
8,14-DihydroxyEFV (8,14-diOH EFV) effect on brain expression of various marker proteins. (**A**), Representative Western blots of brain homogenates. Each lane, except those with molecular weight markers, represents a sample from an individual animal. Of the four samples in each group, two left lanes are always samples from male mice and two right lanes are always samples from female mice. The Western blot for CYP46A1 represents expression in male mice only as a similar gel for female mice is not shown, although quantified in the panel below. Also not shown is a Western blot for CYP46A1 when two female and two male mice for each group were used and the data suggested lack of sex-based differences and the treatment effect. (**B**), Quantification of the relative protein expression in (**A**). Protein expression in each sample within a group was first normalized to the β-actin expression followed by the calculation of the mean value of the protein expression within a group. This mean value was then normalized to the mean value of the protein expression in control 5XFAD mice, which was taken as one. The results represent the mean ± SD of the measurements in individual mice. * *p* ≤ 0.05; ** *p* ≤ 0.01, *** *p* ≤ 0.001 as assessed a two-tailed, unpaired Student’s test. Data for protein expression of EFV-treated mice are shown for comparison and are taken from [7].

Proteins were the same as those assessed previously in EFV-treated mice [7,15]. These were synaptophysin (a marker of synaptic density and presynaptic terminal activity [44,45]), gephyrin (an inhibitory postsynaptic marker [46]), PSD-95 (post synaptic density protein 95, a postsynaptic marker, used frequently to determine the size and strength of synapses [45,47]), GFAP (glial fibrillary acidic protein, a marker for astrocyte activation [48]), Iba1 (ionized calcium-binding adaptor molecule 1, a marker for microglia activation [49]), and CYP46A1. All these proteins were quantified by Western blot in brain homogenates. There did not appear to be sex-based differences in protein levels with the expression of synaptophysin and PSD-95 being increased 1.7- and 1.3-fold, respectively, in 8,14-dihydroxyEFV-treated mice, and the levels of gephyrin remaining unchanged (Figure 8). The expression of GFAP was increased 1.5-fold in the treated group and that of Iba1 was decreased 1.4-fold (Figure 8). The CYP46A1 expression was studied in more detail, i.e., separately for male and female mice (Figure 8) and when samples from both male and female mice were run on the same gel. No changes in the CYP46A1 expression were found between the groups and between sexes within each group. Thus, we obtained evidence that increase in CYP46A1 activity in the treated group was not due to increased protein expression and that as compared to EFV (Figure 8), 8,14-dihydroxyEFV had a similar treatment effect on the expression of PSD-95, GFAP, and Iba1.

## 3. Discussion

One of the major findings of the present work is that the 8,14-dihydroxy metabolite of EFV activated CYP46A1 in mouse brain and elicited similar, but not identical, brain effects as compared to those of the parent drug EFV. 8,14-DihydroxyEFV is a much more polar compound than EFV as indicated by its higher total polar surface area of 78.8 Å^2^ (38.3 Å^2^ for EFV) and a lower predicted log *p* value of 3.17 (4.53 for EFV) [50]. These properties are still within the desirable range for good CNS availability (<90−120 and 2−5, respectively) [51]. Nevertheless, until the present work, it was not clear whether 8,14-dihydroxyEFV indeed crosses the blood–brain barrier and reaches CYP46A1 at the concentration sufficient for enzyme activation. Herein, we demonstrated that 8,14-dihydroxyEFV targets CYP46A1 in vivo, thus suggesting that the blood–brain barrier is permeable to this and other hydroxymetabolites of EFV, which should all be ultimately studied for CYP46A1 activation in vivo.

The 8,14-dihydroxyEFV dose and treatment of 5XFAD mice were the same as with EFV, and the metabolite activated CYP46A1 to a similar extent as EFV (up to ~1.2 fold), despite the maximal CYP46A1 activation by 8,14-dihydroxyEFV in vitro being 1.5-fold higher than that by EFV [50]. To obtain insight into a possible reason for the in vitro–in vivo discrepancy in CYP46A1 activation by 8,14-dihydroxyEFV relative to EFV, we conducted in vitro incubations of purified CYP46A1 either with individual exogenous and endogenous activators or their combination (Figure 7). The data obtained suggested that metabolite binding to the neurotransmitter allosteric site could weaken CYP46A1 activation by 14-dihydroxyEFV in vivo because of a competition with a neurotransmitter (e.g., L-Glu and Ach) for the same binding site. Studies with other hydroxymetabolites of EFV are required to further evaluate the significance of this in vitro insight. In addition, it needs to be ascertained whether: (1) EFV and 8,14-dihydroxyEFV have similar pharmacokinetics, pharmacodynamics, and blood–brain permeability, and (2) the maximal CYP46A1 activation in vivo could only be 1.2-fold due to a strictly controlled cholesterol homeostasis in the brain.

In addition to CYP46A1 activation, there were other 8,14-dihydroxyEFV effects, which were common to those of EFV and were not sex-based. These included increases in cholesterol biosynthesis (increased lathosterol levels, Figure 1), cholesterol turnover (increased lathosterol and 24HC levels, Figure 1), the acetyl-CoA and Ach content (Figure 4), and expression of PSD-95, GFAP, and Iba1. In addition, there was an important common effect, which was observed only in male but not female 8,14-dihydroxyEFV-treated mice—improved performance in the test for the long-term spatial memory (BM, Figure 3). Moreover, 8,14-dihydroxyEFV-treated males had higher increases than female mice in their desmosterol (Figure 1), acetyl-CoA (Figure 4), and Ach (Figure 5) levels. Accordingly, it is tempting to suggest that the sex-based cognitive improvement in 8,14-dihydroxyEFV-treated male mice was determined, at least in part, by the desmosterol-acetyl-CoA-Ach-memory axis. Possibly, this axis was mainly operative in the hippocampus as learning improvement was only observed in the BM test, a hippocampal-dependent task [52], and hippocampus is one of the most affected brain area in 5XFAD mice [14].

Desmosterol is a marker of cholesterol biosynthesis in astrocytes [28] and an activating ligand for liver X receptors [53]. The latter are transcription factors that integrate many biological processes including cholesterol and glucose metabolism as well as immune and inflammatory responses along with apoptosis and phagocytosis [54,55]. Remarkably, of the LXR-regulated processes, glucose metabolism represents the major source of acetyl-CoA in the brain [56]. We found that glucose metabolism was affected in EFV-treated mice [32] and linked the increased production of acetyl-CoA to increased production of Ach (Figure 5 and Figure 6). The Ach levels are decreased in AD because of selective degeneration of acetylcholine-releasing neurons in the basal forebrain, whose cell bodies provide widespread innervation of the cerebral cortex and related structures. As such, Ach plays an important role in cognitive functions, especially memory [57].

The caveat to the desmosterol-memory axis is that an increase in the Ach levels in 8,14-dihydroxyEFV-treated female mice did not lead to memory improvement (Figure 3 and Figure 5), possibly because this increase was not sufficient. In addition, EFV-treated mice had a lower increase in Ach levels (Figure 5), but nevertheless, both sexes showed improved learning in the Morris water maze test and contextual fear conditioning test [7]. Apparently, brain Ach levels are not the only factors that affect behavioral performance in EFV and 8,14-dihydroxyEFV-treated mice.

Despite the caveats, our data showing that increased levels of acetyl-CoA lead to increased Ach production and differential upregulation of the Ach-related genes in 8,14-dihydroxyEFV-treated animals (Figure 6) are important and novel findings. First, they support the therapeutic potential of EFV and 8,14-dihydroxyEFV as anti-AD therapeutics. Currently, Ach decrease in AD is mitigated by the ACHE inhibitors donepezil, galantamine, and rivastigmine, all of which target Ach elimination and are approved by the FDA for AD symptomatic treatment [58]. EFV and 8,14-dihydroxyEFV increase Ach levels by a different mechanism, namely via increased production. Hence, a simultaneous administration of EFV or 8,14-dihydroxyEFV and an ACHE inhibitor should have a synergistic effect and increase Ach production. Second, our data expand the knowledge of the beneficial CYP46A1 activation effects. CYP46A1 is emerging as a key brain enzyme, which controls multiple, apparently unlinked, brain processes either directly (via cholesterol elimination) or indirectly (via the mevalonate and acetyl-CoA production and physico-chemical properties of plasma membranes [1,2,27,32,59]. Specific direct and indirect CYP46A1 activity-dependent effects are still under investigation, including the present work, in which we identified a new process, the Ach production, affected by CYP46A1 activation.

A decrease in the amount of insoluble Aβ_42_ peptide in brain homogenates was the only in vivo effect that was more pronounced in the treatment with 8,14-dihydroxyEFV than EFV (Figure 3). This decrease was not sex-specific and was not observed in EFV-treated 5XFAD mice, which only showed region-specific decreases in the Aβ burden [7]. Data have emerged that extend Ach and cholinergic innervation beyond synaptic transmission. These include stimulation by Ach of the non-amyloidogenic cleavage of amyloid precursor protein and thereby inhibition of the Aβ production as well as control of tau phosphorylation [60]. A decrease in the Aβ_42_ peptide levels in 8,14-dihydroxyEFV-treated female and male mice (Figure 2) supports the Ach roles beyond neuromodulation and is in agreement with an increase in the brain Ach levels (Figure 5). Further research is needed to support the Ach-Aβ_42_ peptide connection in 8,14-dihydroxyEFV-treated mice.

The sex-independent decrease of the Aβ pathology was the major clinically relevant positive effect of 8,14-dihydroxyEFV, whereas that of EFV was on cognitive function [7]. As 8,14-dihydroxyEFV is only barely detectable in human plasma [20], a study in which this metabolite is co-administered with EFV to 5XFAD mice is thus warranted to ascertain whether there will be simultaneous positive effects on Aβ burden and learning performance. The underlying reasons for the differential 8,14-dihydroxyEFV and EFV effects also need to be clarified.

In summary, to further characterize EFV as an anti-AD therapeutic and to identify more potent in vivo activators of CYP46A1 than EFV, we focused on 8,14-dihydroxyEFV, the phase 1 minor metabolite of EFV. We found that many of the metabolite brain effects were common with those of EFV and observed either in 5XFAD mice of both sexes or only in male animals. The common sex-independent effects were activation of CYP46A1 and brain cholesterol turnover, increases in acetyl-CoA and Ach levels, and changes in PSD-95, GFAP, and Iba1 expression. The common sex-dependent effect (only in male mice) was improved performance in the BM test, which, along with other male-specific effects of 8,14-dihydroxyEFV, suggested the desmosterol-acetyl-CoA-Ach-cognition axis. A positive difference between 8,14-dihydroxyEFV and EFV was that the former reduced the Aβ_42_ levels in brain homogenates. The present work provides clear directions for our future studies of EFV and its metabolites and warrants a treatment in which EFV should be co-administered to 5XFAD mice with 8,14-dihydroxyEFV.

## 4. Materials and Methods

### 4.1. Animals and Treatment

5XFAD mice hemizygous for the mutant (K670N, M671L, I716V, V717I) human amyloid precursor protein 695 and mutant (M146L and L286V) human presenilin 1 [14] were generated as described [6]. Briefly, hemizygous male mice (the Jackson Laboratory, stock No: 34840, Bar Harbor, ME, USA, on the B6SJL background, Bar Harbor, ME, United States) were crossed with wild type B6SJL females (the Jackson Laboratory, stock No: 100012), which were free of the *Pde6b^rd1^* mutation leading to early onset severe retinal degeneration and blindness [61]. The *Pde6b^rd1^* mutation was bred out of female B6SJL mice. Only the F1 generation was used. Animal treatment with 14-dihydroxyEFV (racemic mixture, Toronto Research Chemicals, #D452800, Toronto, ON, Canada) was the same as with EFV [7], i.e., 8,14-dihydroxyEFV was administered in drinking water containing 0.0004% Tween 80 at a 0.1 mg/kg body weight/day dose from 3 to 9 months of age. Control animals received aqueous 0.0004% Tween 80. Mice were maintained in a temperature- and humidity-controlled environment with 12 h light/12 h dark cycle in cages with water and food ad libitum. All animal experiments were approved by the Case Western Reserve University’s Institutional Animal Care and Use Committee and conformed to recommendations by the American Veterinary Association Panel on Euthanasia.

### 4.2. Behavioral Assessments

Experiments were performed in the following order: the BM test at days 1–3 and 5; the Y-maze test at day 8; and the fear conditioning memory tests at days 9–10. The schedule for the BM test was as follows. Day 1 was acclimation to the BM apparatus. Mice were placed under a glass beaker near the designated escape hole and allowed 3 min to voluntarily enter the hole. Mice could view the hole and its relation to visual cues around the maze. If the mouse did not enter the hole during that time period, it was gently placed into the hole for 1 min prior to returning to their home cage. Days 2 and 3 were the training sessions 1–3 and 4–5, respectively. Mice were placed into the maze and were given a maximum of 2 min to find the escape hole assigned during the acclimation period. During day 2, mice explored the maze using visual cues on the wall and floor of the maze to locate the escape hole and escape a brightly lit arena. After 2 min, mice were placed near the hole in the same location as day 1 habituation for 1 min. If the mouse failed to voluntarily enter the hole, the animal was gently placed in the hole for 1 min and then returned to their home cage. This was repeated 3 times with a minimum of 1 h intertrial interval. During day 3, the procedure was the same as for day 2 with training being repeated two times and a minimum of 1 h intertrial.

In the Y-maze test, mice were allowed 8 min of exploration in a y-shaped maze with equal length arms, where the number of entries, order of entries (Arms A, B, and C), and spontaneous alterations (entry into three arms non-consecutively, for example, A-B-C, B-C-A, C-A-B equals 3 spontaneous alterations) were recorded. These data were also used to calculate the percentage of entries that were spontaneous alterations.

In the contextual and cued fear memory tests, mice were placed in a chamber and presented with one 30-s audible tone that co-terminated with a mild foot shock. After the tone shock pairing, mice were returned to their home cage for 24 h prior to returning to the same unaltered chamber for 5 min of observation (contextual memory). Five hours later, the mice were returned to the chamber with altered tactile, visual, and olfactory cues (cued memory). In the altered chamber, mice were presented with two audible tones identical to the tones presented during the initial tone/shock pairing. The behavior of interest was freezing, which was defined as the cessation of all movement except for breathing. Freezing was used to index fear memory behavior, which was presented as a percent of freezing time during the experiment.

### 4.3. Brain Processing

This was as described after overnight fasting [6,16,62]. Briefly, the brains were isolated, rinsed in cold phosphate buffer saline, and blotted. The brain stem, cerebellum, and olfactory bulb were removed, and the brain was dissected along the midline to obtain two hemispheres. One randomly selected hemisphere was used for the brain homogenate (10%, *w*/*v*) preparation using 50 mM potassium phosphate buffer (KP_i_), pH 7.2, containing 300 mM sucrose, 0.5 mM dithiothreitol, 1 mM EDTA, and a cocktail of protease inhibitors (cOmplete, Sigma-Aldrich, #11697498001, St. Louis, MO, USA). The brain homogenates were then used for sterol, Aβ, acetyl-CoA, and Ach quantifications as well as Western blots. The other hemisphere was homogenized in 1 mL of the TRIzol™ Reagent (Thermo Fisher Scientific, #15596026, Waltham, MA, USA) and was used for total RNA isolation according to the manufacturer’s instructions.

### 4.4. Quantitative Studies

Either free or total sterol content (a sum of free and esterified sterol) was measured as described [63] using isotope dilution gas chromatography-mass spectrometry and deuterated sterol analogs as internal standards.

Soluble and insoluble Aβ peptides were extracted with 0.2% diethylamine and 70% formic acid as described [6] and quantified by ELISA kits for Aβ_40_ and Aβ_42_ (Thermo Fisher Scientific, #KHB3482 and #KHB3441, respectively) according to the manufacturer’s instructions.

The acetyl-CoA content was measured using the Acetyl-Coenzyme A assay kit (Sigma-Aldrich, #MAK039) in brain homogenates and mitochondria, which were prepared as described [32,64]. Briefly, the mitochondria isolation included the resuspension of the 21,000 g pellet in 1 mL of 15% Percoll and subsequent centrifugation on the Percoll density gradient (23–40%) at 30,700× *g* and 4 °C for 5 min. The band at the interface of 23% and 40% Percoll was removed and mixed with the isolation buffer (10 mM Tris, pH 7.4, 166 mM sucrose, and 1 mM EDTA) containing 0.02% digitonin. The mitochondria were obtained after centrifugation at 16,700× *g* and 4 °C for 10 min, the resuspension of the pellet in the isolation buffer and another centrifugation (6900× *g*, 4 °C, 10 min). Mitochondrial fractions (the pellet) were assessed by Western blot using ATP5A (ATP synthase F1 subunit α) and calnexin as markers for mitochondria and endoplasmic reticulum, respectively [32]. The relative intensity of the anti-ATP5A and anti-calnexin signals was 99% and 1%, respectively.

Free and total choline were also quantified by a kit (LifeSpan BioSciences, Inc., LS-K119, Seattle, WA, USA) according to the manufacturer’s instructions.

Western blots were carried out as described [7,16]. The primary and secondary antibodies are summarized in Supplementary Table S1.

For the measurements of gene expression, total RNA (1 μg) was converted to cDNA by SuperScript III Reverse Transcriptase (Thermo Fisher Scientific, #18080044) according to the manufacturer’s protocol. PCR reactions (performed in triplicate for each gene and animal sample) were carried out as described [15] using 2 μL of cDNA, a pair of gene-specific primers (Supplementary Table S2), and a FastStart Universal SYBR Green Master (Rox) (Sigma-Aldrich, #4913850001). Changes in the relative mRNA levels were calculated by the 2^−ΔΔCt^ method [65] after the normalization of gene expression to the expression of *Gapdh*.

### 4.5. CYP46A1 Activation In Vitro

Human truncated Δ(2–50) CYP46A1 with a four-histidine tag on the C terminus and rat cytochrome P450 oxidoreductase were expressed in *Escherichia coli* and purified as described [66,67]. Enzyme assays were carried out as described [50] in 1 mL of 50 mM KP_i_ buffer (pH 7.2) containing 100 mM NaCl, 40 μg/mL L-α-1,2-dilauroyl-*sn*-glycero-3-phosphocholine, 0.5 µM CYP46A1, 1.0 µM cytochrome P450 oxidoreductase, 40 µM cholesterol, 2 units of catalase, and an NADPH-regenerating system (1 mM NADPH, 10 mM glucose-6-phosphate, and 2 units of glucose-6-phosphate dehydrogenase). If activators were included, their concentrations were 20 µM for EFV and 8,14-dihydroxyEFV and 100 µM for Ach as at these concentrations, the maximal CYP46A1 activation is observed in the presence of 20–40 µM cholesterol [17,19,25]. The reaction time was 30 min at 37 °C, and sterol extraction was with 5 mL of dichloromethane containing 3 nmol of 24-hydroxy-[25,26,26,26,27,27,27-^2^H_7_]-cholesterol, which served as an internal standard. Sterol extracts were then processed and analyzed by gas chromatography-mass spectrometry as described [63].

### 4.6. Statistics

Data from all available animals were used. There was no exclusion of statistical outliers. The sample size (n) and statistical analysis are indicated in each figure or figure legend. All data represent the mean ± SD of the measurements in individual animals, except behavioral tests where the mean ± SEM was used. All Western blots were repeated at least 2 times. Statistical analyses were either by unpaired Student’s *t* test assuming a 2-tailed distribution or two-way repeated measures ANOVA with Tukey’s multiple comparisons, or two-way repeated measures ANOVA with Bonferroni correction. Statistical significance was defined as * *p* ≤ 0.05; ** *p* ≤ 0.01; *** *p* ≤ 0.001.

## Data Availability

The data presented in this study are available within the article and Supplementary Materials.

## Supplementary Materials

The following supporting information can be downloaded at: https://www.mdpi.com/article/10.3390/ijms23147669/s1.

## References

[1] Pikuleva I.A., Cartier N. (2021). Cholesterol Hydroxylating Cytochrome P450 46A1: From Mechanisms of Action to Clinical Applications. Front. Aging Neurosci..

[2] Pikuleva I.A. (2021). Targeting cytochrome P450 46A1 and brain cholesterol 24-hydroxylation to treat neurodegenerative diseases. Explor. Neuroprotective Ther..

[3] Hudry E., Van Dam D., Kulik W., De Deyn P.P., Stet F.S., Ahouansou O., Benraiss A., Delacourte A., Bougneres P., Aubourg P. (2010). Adeno-associated virus gene therapy with cholesterol 24-hydroxylase reduces the amyloid pathology before or after the onset of amyloid plaques in mouse models of Alzheimer’s disease. Mol. Ther. J. Am. Soc. Gene Ther..

[4] Burlot M.A., Braudeau J., Michaelsen-Preusse K., Potier B., Ayciriex S., Varin J., Gautier B., Djelti F., Audrain M., Dauphinot L. (2015). Cholesterol 24-hydroxylase defect is implicated in memory impairments associated with Alzheimer-like Tau pathology. Hum. Mol. Genet..

[5] Boussicault L., Alves S., Lamaziere A., Planques A., Heck N., Moumne L., Despres G., Bolte S., Hu A., Pages C. (2016). CYP46A1, the rate-limiting enzyme for cholesterol degradation, is neuroprotective in Huntington’s disease. Brain.

[6] Mast N., Saadane A., Valencia-Olvera A., Constans J., Maxfield E., Arakawa H., Li Y., Landreth G., Pikuleva I.A. (2017). Cholesterol-metabolizing enzyme cytochrome P450 46A1 as a pharmacologic target for Alzheimer’s disease. Neuropharmacology.

[7] Petrov A.M., Lam M., Mast N., Moon J., Li Y., Maxfield E., Pikuleva I.A. (2019). CYP46A1 Activation by Efavirenz Leads to Behavioral Improvement without Significant Changes in Amyloid Plaque Load in the Brain of 5XFAD Mice. Neurother. J. Am. Soc. Exp. NeuroTherapeutics.

[8] Patel T.K., Patel V.B., Rana D.G. (2017). Possible anti-depressant effect of efavirenz and pro-depressive-like effect of voriconazole in specified doses in various experimental models of depression in mice. Pharmacol. Rep..

[9] Kacher R., Lamaziere A., Heck N., Kappes V., Mounier C., Despres G., Dembitskaya Y., Perrin E., Christaller W., Sasidharan Nair S. (2019). CYP46A1 gene therapy deciphers the role of brain cholesterol metabolism in Huntington’s disease. Brain.

[10] Mitroi D.N., Pereyra-Gomez G., Soto-Huelin B., Senovilla F., Kobayashi T., Esteban J.A., Ledesma M.D. (2019). NPC1 enables cholesterol mobilization during long-term potentiation that can be restored in Niemann-Pick disease type C by CYP46A1 activation. EMBO Rep..

[11] Nobrega C., Mendonca L., Marcelo A., Lamaziere A., Tome S., Despres G., Matos C.A., Mechmet F., Langui D., den Dunnen W. (2019). Restoring brain cholesterol turnover improves autophagy and has therapeutic potential in mouse models of spinocerebellar ataxia. Acta Neuropathol..

[12] Han M., Wang S., Yang N., Wang X., Zhao W., Saed H.S., Daubon T., Huang B., Chen A., Li G. (2020). Therapeutic implications of altered cholesterol homeostasis mediated by loss of CYP46A1 in human glioblastoma. EMBO Mol. Med..

[13] Ali T., Hannaoui S., Nemani S., Tahir W., Zemlyankina I., Cherry P., Shim S.Y., Sim V., Schaetzl H.M., Gilch S. (2021). Oral administration of repurposed drug targeting Cyp46A1 increases survival times of prion infected mice. Acta Neuropathol. Commun..

[14] Oakley H., Cole S.L., Logan S., Maus E., Shao P., Craft J., Guillozet-Bongaarts A., Ohno M., Disterhoft J., Van Eldik L. (2006). Intraneuronal beta-amyloid aggregates, neurodegeneration, and neuron loss in transgenic mice with five familial Alzheimer’s disease mutations: Potential factors in amyloid plaque formation. J. Neurosci..

[15] Petrov A.M., Mast N., Li Y., Pikuleva I.A. (2019). The key genes, phosphoproteins, processes, and pathways affected by efavirenz-activated CYP46A1 in the amyloid-decreasing paradigm of efavirenz treatment. FASEB J..

[16] Mast N., El-Darzi N., Petrov A.M., Li Y., Pikuleva I.A. (2020). CYP46A1-dependent and independent effects of efavirenz treatment. Brain Commun..

[17] Mast N., Li Y., Linger M., Clark M., Wiseman J., Pikuleva I.A. (2014). Pharmacologic stimulation of cytochrome P450 46A1 and cerebral cholesterol turnover in mice. J. Biol. Chem..

[18] Anderson K.W., Mast N., Hudgens J.W., Lin J.B., Turko I.V., Pikuleva I.A. (2016). Mapping of the allosteric site in cholesterol hydroxylase CYP46A1 for efavirenz, a drug that stimulates enzyme activity. J. Biol. Chem..

[19] Mast N., Anderson K.W., Johnson K.M., Phan T.T.N., Guengerich F.P., Pikuleva I.A. (2017). In Vitro cytochrome P450 46A1 (CYP46A1) activation by neuroactive compounds. J. Biol. Chem..

[20] Avery L.B., VanAusdall J.L., Hendrix C.W., Bumpus N.N. (2013). Compartmentalization and antiviral effect of efavirenz metabolites in blood plasma, seminal plasma, and cerebrospinal fluid. Drug Metab. Dispos..

[21] Ward B.A., Gorski J.C., Jones D.R., Hall S.D., Flockhart D.A., Desta Z. (2003). The cytochrome P450 2B6 (CYP2B6) is the main catalyst of efavirenz primary and secondary metabolism: Implication for HIV/AIDS therapy and utility of efavirenz as a substrate marker of CYP2B6 catalytic activity. J. Pharmacol. Exp. Ther..

[22] Ogburn E.T., Jones D.R., Masters A.R., Xu C., Guo Y., Desta Z. (2010). Efavirenz primary and secondary metabolism in vitro and in vivo: Identification of novel metabolic pathways and cytochrome P450 2A6 as the principal catalyst of efavirenz 7-hydroxylation. Drug Metab. Dispos..

[23] Bumpus N.N., Kent U.M., Hollenberg P.F. (2006). Metabolism of efavirenz and 8-hydroxyefavirenz by P450 2B6 leads to inactivation by two distinct mechanisms. J. Pharmacol. Exp. Ther..

[24] Avery L.B., Sacktor N., McArthur J.C., Hendrix C.W. (2013). Protein-free efavirenz concentrations in cerebrospinal fluid and blood plasma are equivalent: Applying the law of mass action to predict protein-free drug concentration. Antimicrob. Agents Chemother..

[25] Mast N., Fotinich A., Pikuleva I.A. (2022). The hydroxylation position rather than chirality determines how efavirenz metabolites activate CYP46A1 In Vitro. Drug Metab Dispos.

[26] Dietschy J.M., Turley S.D. (2001). Cholesterol metabolism in the brain. Curr. Opin. Lipidol..

[27] Lund E.G., Xie C., Kotti T., Turley S.D., Dietschy J.M., Russell D.W. (2003). Knockout of the cholesterol 24-hydroxylase gene in mice reveals a brain-specific mechanism of cholesterol turnover. J. Biol. Chem..

[28] Pfrieger F.W., Ungerer N. (2011). Cholesterol metabolism in neurons and astrocytes. Prog. Lipid Res..

[29] Murphy M.P., LeVine H. (2010). Alzheimer’s disease and the amyloid-beta peptide. J. Alzheimer’s Dis..

[30] Haass C., Selkoe D.J. (2007). Soluble protein oligomers in neurodegeneration: Lessons from the Alzheimer’s amyloid beta-peptide. Nat. Rev. Mol. Cell. Biol..

[31] Pietrocola F., Galluzzi L., Bravo-San Pedro J.M., Madeo F., Kroemer G. (2015). Acetyl coenzyme A: A central metabolite and second messenger. Cell Metab.

[32] Mast N., Petrov A.M., Prendergast E., Bederman I., Pikuleva I.A. (2021). Brain Acetyl-CoA Production and Phosphorylation of Cytoskeletal Proteins Are Targets of CYP46A1 Activity Modulation and Altered Sterol Flux. Neurother. J. Am. Soc. Exp. NeuroTherapeutics.

[33] Tucek S. (1993). Short-term control of the synthesis of acetylcholine. Prog. Biophys. Mol. Biol..

[34] Chen Z.R., Huang J.B., Yang S.L., Hong F.F. (2022). Role of Cholinergic Signaling in Alzheimer’s Disease. Molecules.

[35] Terry A.V., Buccafusco J.J. (2003). The cholinergic hypothesis of age and Alzheimer’s disease-related cognitive deficits: Recent challenges and their implications for novel drug development. J. Pharmacol. Exp. Ther..

[36] Okuda T., Haga T. (2000). Functional characterization of the human high-affinity choline transporter. FEBS Lett..

[37] Okuda T., Haga T., Kanai Y., Endou H., Ishihara T., Katsura I. (2000). Identification and characterization of the high-affinity choline transporter. Nat. Neurosci..

[38] Augustinsson K.B., Nachmansohn D. (1949). Distinction between Acetylcholine-Esterase and Other Choline Ester-splitting Enzymes. Science.

[39] Ahmed T., Zahid S., Mahboob A., Farhat S.M. (2017). Cholinergic System and Post-translational Modifications: An Insight on the Role in Alzheimer’s Disease. Curr. Neuropharmacol..

[40] Erickson J.D., Varoqui H., Schafer M.K., Modi W., Diebler M.F., Weihe E., Rand J., Eiden L.E., Bonner T.I., Usdin T.B. (1994). Functional identification of a vesicular acetylcholine transporter and its expression from a "cholinergic" gene locus. J. Biol. Chem..

[41] Maeda S., Qu Q., Robertson M.J., Skiniotis G., Kobilka B.K. (2019). Structures of the M1 and M2 muscarinic acetylcholine receptor/G-protein complexes. Science.

[42] Perrier A.L., Massoulie J., Krejci E. (2002). PRiMA: The membrane anchor of acetylcholinesterase in the brain. Neuron.

[43] Oda Y. (1999). Choline acetyltransferase: The structure, distribution and pathologic changes in the central nervous system. Pathol. Int..

[44] Kwon S.E., Chapman E.R. (2011). Synaptophysin regulates the kinetics of synaptic vesicle endocytosis in central neurons. Neuron.

[45] Nithianantharajah J., Levis H., Murphy M. (2004). Environmental enrichment results in cortical and subcortical changes in levels of synaptophysin and PSD-95 proteins. Neurobiol. Learn. Mem..

[46] Essrich C., Lorez M., Benson J.A., Fritschy J.-M., Lüscher B. (1998). Postsynaptic clustering of major GABAA receptor subtypes requires the γ2 subunit and gephyrin. Nat. Neurosci..

[47] Holtmaat A., Svoboda K. (2009). Experience-dependent structural synaptic plasticity in the mammalian brain. Nat. Rev. Neurosci..

[48] Jurga A.M., Paleczna M., Kadluczka J., Kuter K.Z. (2021). Beyond the GFAP-Astrocyte Protein Markers in the Brain. Biomolecules.

[49] Schwabenland M., Brück W., Priller J., Stadelmann C., Lassmann H., Prinz M. (2021). Analyzing microglial phenotypes across neuropathologies: A practical guide. Acta Neuropathol..

[50] Mast N., Verwilst P., Wilkey C.J., Guengerich F.P., Pikuleva I.A. (2020). In Vitro Activation of Cytochrome P450 46A1 (CYP46A1) by Efavirenz-Related Compounds. J. Med. Chem..

[51] Hitchcock S.A., Pennington L.D. (2006). Structure-brain exposure relationships. J. Med. Chem..

[52] Bach M.E., Hawkins R.D., Osman M., Kandel E.R., Mayford M. (1995). Impairment of spatial but not contextual memory in CaMKII mutant mice with a selective loss of hippocampal LTP in the range of the theta frequency. Cell.

[53] Spann N.J., Garmire L.X., McDonald J.G., Myers D.S., Milne S.B., Shibata N., Reichart D., Fox J.N., Shaked I., Heudobler D. (2012). Regulated accumulation of desmosterol integrates macrophage lipid metabolism and inflammatory responses. Cell.

[54] Calkin A.C., Tontonoz P. (2012). Transcriptional integration of metabolism by the nuclear sterol-activated receptors LXR and FXR. Nat. Rev. Mol. Cell. Biol..

[55] Jakobsson T., Treuter E., Gustafsson J.A., Steffensen K.R. (2012). Liver X receptor biology and pharmacology: New pathways, challenges and opportunities. Trends Pharmacol. Sci..

[56] Ronowska A., Szutowicz A., Bielarczyk H., Gul-Hinc S., Klimaszewska-Łata J., Dyś A., Zyśk M., Jankowska-Kulawy A. (2018). The Regulatory Effects of Acetyl-CoA Distribution in the Healthy and Diseased Brain. Front. Cell. Neurosci..

[57] Coyle J.T., Price D.L., DeLong M.R. (1983). Alzheimer’s disease: A disorder of cortical cholinergic innervation. Science.

[58] Li X.T. (2022). Alzheimer’s disease therapy based on acetylcholinesterase inhibitor/blocker effects on voltage-gated potassium channels. Metab. Brain Dis..

[59] Petrov A.M., Mast N., Li Y., Denker J., Pikuleva I.A. (2020). Brain sterol flux mediated by cytochrome P450 46A1 affects membrane properties and membrane-dependent processes. Brain Commun..

[60] Ovsepian S.V., O’Leary V.B., Zaborszky L. (2016). Cholinergic Mechanisms in the Cerebral Cortex: Beyond Synaptic Transmission. Neuroscientist.

[61] Chang B., Hawes N.L., Hurd R.E., Davisson M.T., Nusinowitz S., Heckenlively J.R. (2002). Retinal degeneration mutants in the mouse. Vision Res..

[62] Petrov A.M., Pikuleva I.A. (2019). Cholesterol 24-Hydroxylation by CYP46A1: Benefits of Modulation for Brain Diseases. Neurother. J. Am. Soc. Exp. NeuroTherapeutics.

[63] Mast N., Reem R., Bederman I., Huang S., DiPatre P.L., Björkhem I., Pikuleva I.A. (2011). Cholestenoic acid is an important elimination product of cholesterol in the retina: Comparison of retinal cholesterol metabolism with that in the brain. Investig. Ophthalmol. Vis. Sci..

[64] Sims N.R., Anderson M.F. (2008). Isolation of mitochondria from rat brain using Percoll density gradient centrifugation. Nat. Protoc..

[65] Pfaffl M.W. (2001). A new mathematical model for relative quantification in real-time RT-PCR. Nucleic Acids Res..

[66] White M.A., Mast N., Björkhem I., Johnson E.F., Stout C.D., Pikuleva I.A. (2008). Use of complementary cation and anion heavy-atom salt derivatives to solve the structure of cytochrome P450 46A1. Acta Crystallogr. D Biol. Crystallogr..

[67] Hanna I.H., Teiber J.F., Kokones K.L., Hollenberg P.F. (1998). Role of the alanine at position 363 of cytochrome P450 2B2 in influencing the NADPH- and hydroperoxide-supported activities. Arch. Biochem. Biophys..

