# Supplementary material for: Increased Acetylcholine Levels and Other Brain Effects in 5XFAD Mice after Treatment with 8,14-Dihydroxy Metabolite of Efavirenz
Source: Int J Mol Sci. 2022 Jul 11;23(14):7669. doi: 10.3390/ijms23147669 (PMC9317559; doi:10.3390/ijms23147669)
Supplement: Supplementary file 1 [file ijms-23-07669-s001.zip › ijms-1815454-supplementary.pdf]

Supplementary Materials

Increased acetylcholine levels and other brain effects in 5XFAD mice after treatment with 8,14-dihydroxy metabolite of efavirenz

Natalia Mast, Young Li, and Irina A. Pikuleva

Department of Ophthalmology and Visual Sciences, Case Western Reserve University, Cleveland, OH 44106, USA

Supplementary Table S1. Antibodies Used for Western Blots

Antigen	Primary antibody		Secondary antibody	
	Clonality, host, dilution	Catalog #, vendor	Clonality, host, dilution	Catalog #, vendor
PSD-95	Monoclonal mouse IgG, 1:4,000	ab2723, Abcam, Cambridge, MA	Goat anti-mouse IgG Birdie 680RD, 1:10,000	925-68070, Li-Cor, Lincoln, NE
Gephyrin	Monoclonal mouse IgG, 1:2,000	147111, Synaptic Systems, Goettingen, Germany		
Synapto-physin	Monoclonal mouse IgG, 1:4,000	101011, Synaptic Systems, Goettingen, Germany		
CYP46A1	Monoclonal mouse IgG (clone 9C5-B2-A1), 1:500	Hybridoma facility at Visual Sciences Research Center, Case Western Reserve University		
ATP5A	Monoclonal mouse IgG, 1:1,000	ab14748, Abcam, Cambridge, MA		
GFAP	Polyclonal chicken IgG, 1:5,000	PA1-10004, Thermo Fisher, Waltham, MA	Donkey anti-chicken IgG IRDye 680RD, 1:10,000	926-68075, Li-Cor, Lincoln, NE
Iba1	Monoclonal rabbit IgG, 1:2,000	ab178846, Abcam, Cambridge, MA	Goat anti-rabbit IgG IRDye 800 CW, 1:10,000	925-32211, Li-Cor, Lincoln, NE
Calnexin	Polyclonal rabbit IgG, 1:1,000	ab22595, Abcam, Cambridge, MA		
β-Actin	Polyclonal mouse IgG, 1:2,000	ab8226, Abcam, Cambridge, MA	Goat anti-mouse IgG IRDye 680RD, 1:10,000	925-68070, Li-Cor, Lincoln, NE
β-Actin	Polyclonal rabbit IgG, 1:1,000	ab8227, Abcam, Cambridge, MA	Goat anti-rabbit IgG IRDye 800 CW, 1:10,000	925-32211, Li-Cor, Lincoln, NE

Supplementary Table S2. List of Primers for Quantitative Real-Time PCR.

Gene	Forward primer	Reverse primer
Ache	5'-GAGATGCCATGAGTGCAGTG-3'	5'-GGCACGGTGTTCAAAGATGT-3'
Chat	5'-AACCTGGCTACCTTCCAGTC-3'	5'-CTAGCAACCAGGCAGTCAAC-3'
Chrm1	5'-GCCTCCTGTCTTCAGTGTCT-3'	5'-ATTACCTGTGGCAGCCTCTT-3'
Chrm2	5'-CCTGTAGTGTGCGACCTTTG-3'	5'-CAGCTGCAATCATCATGCCT-3'
Chrna4	5'-TTGGCTTGTCGATTGCTCAG-3'	5'-CGCAGTTTGTAGTCATGCCA-3'
Chrna7	5'-CCCTGGCTTTGCTGGTATTC-3'	5'-GCACGGAATCAGATGTTGCT-3'
Gapdh	5'-AGTCCATGCCATCACTGCCACC -3'	5'-CCAGTGAGCTTCCCGTTCAGC -3'
Gna11	5'-CATAGTCACTGCACCTTGCC-3'	5'-CCTGCCTATGTCCTCTCTGG-3'
Prima1	5'-AGGCAAGTTAGCTCACCAGT-3'	5'-AGCGTGTGTCACCTGAACTA-3'
Slc5a7	5'-TCACTGGCTAATGCACTGGA-3'	5'-GCAAGAGCAAGACAGCCTAC-3'
Slc18a3	5'-AGCCCTACCCTGATCTCTGA-3'	5'-CAAGTAGGCGCTGGCATTAG-3'
